# Cytidine-5-diphosphocholine reduces microvascular permeability during experimental endotoxemia

**DOI:** 10.1186/s12871-015-0086-9

**Published:** 2015-08-01

**Authors:** Karsten Schmidt, Jochen Frederick Hernekamp, Miriam Doerr, Aleksandar R. Zivkovic, Thorsten Brenner, Andreas Walther, Markus A. Weigand, Stefan Hofer

**Affiliations:** 1 Department of Anesthesiology, Heidelberg University Hospital, Im Neuenheimer Feld 110, 69120 Heidelberg, Germany; 2 Department of Hand, Plastic and Reconstructive Surgery, Burn Center, BG Trauma Center Ludwigshafen, Ludwigshafen, University of Heidelberg, Heidelberg, Germany; 3 Department of General, Visceral and Transplant Surgery, University of Heidelberg, Heidelberg, Germany; 4 Department of Anesthesiology, Katharinenhospital, Stuttgart, Germany

**Keywords:** Endotoxemia, CDP-choline, Endothelial dysfunction, Microvascular permeability, Intravital microscopy

## Abstract

**Background:**

Microvascular permeability and leukocyte adhesion are pivotal mechanisms in sepsis pathophysiology contributing to the development of shock and mortality. No effective pharmacological therapy is currently available to restore microvascular barrier function in sepsis. Cholinergic mediators have been demonstrated to exert anti-inflammatory effects during inflammation. Cytidine-5-diphosphocholine (CDP-choline) is an extensively studied cholinergic drug due to its brain protective characteristics in cerebrovascular diseases. This study evaluated the effect of CDP-choline on microvascular permeability and leukocyte adhesion during endotoxemia.

**Methods:**

Macromolecular leakage, leukocyte adhesion, and venular wall shear rate were examined in mesenteric postcapillary venules of rats by using intravital microscopy (IVM). Lipopolysaccharide (LPS) (4 mg/kg/h) or equivalent volumes of saline were continuously infused following baseline IVM at 0 min. IVM was repeated after 60 and 120 min in endotoxemic and nonendotoxemic animals. CDP-choline (100 mg/kg) was applied as an i.v. bolus. Animals received either saline alone, CDP-choline alone, CDP-choline 10 min before or 30 min after LPS administration, or LPS alone. Due to nonparametric data distribution, Wilcoxon test and Dunn's multiple comparisons test were used for data analysis. Data were considered statistically significant at *p* < 0.05.

**Results:**

Treatment with LPS alone significantly increased microvascular permeability and leukocyte adhesion and decreased venular wall shear rate. CDP-choline significantly reduced microvascular permeability in animals treated with LPS. Leukocyte adhesion and venular wall shear rate were not affected by CDP-choline during endotoxemia.

**Conclusion:**

CDP-choline has a protective effect on microvascular barrier function during endotoxemia. Considering the excellent pharmacologic safety profile of CDP-choline, its use could be an approach for the treatment of capillary leakage in sepsis.

## Background

Sepsis is a serious problem in critical care medicine with significant mortality [1] directly associated to microcirculatory alterations [2]. Microcirculatory alterations are early indicators of tissue injury prior to septic organ failure, characterized by the functional breakdown of microvascular perfusion with concomitant tissue edema formation. Progressive edema formation due to increased endothelial permeability leads to impaired tissue oxygenation, which precedes organ damage and shock in sepsis.

Inflammatory endothelial cell activation and leukocyte-endothelial interactions can be positively influenced by cholinergic mediators [3, 4]. This beneficial cholinergic anti-inflammatory effect on endothelial function can be attributed to the activation of anti-inflammatory neuro-immunological mechanisms described by Tracey et al. [5, 6]. The inflammatory reflex and the cholinergic anti-inflammatory pathway (CAP) modulate the magnitude of the innate immune response by limiting pro-inflammatory processes to a non-toxic range, thereby minimizing tissue injury [5, 6]. CAP-mediated anti-inflammatory signals result in the release of acetylcholine (ACh), which interacts with innate immune cells that express the nicotinic acetylcholine receptor subunit α7 (α7nAChR). Intracellular α7nAChR signal transduction inhibits the transcription of pro-inflammatory genes [5, 6]. Human and rat endothelial cells express α7nAChR, identifying the endothelium as a target for anti-inflammatory cholinergic mediators [3, 7].

Cytidine-5-diphosphocholine (CDP-choline) has been studied for its brain protective properties in cerebrovascular and neurodegenerative diseases in several clinical trials, and has shown an excellent drug safety profile [8–10].

Locally administered CDP-choline reduced TNF alpha production and tissue edema in a carrageenan-induced inflammatory pain model dependent on the presence of inflammation and non-neuronal α7nAChR [11]. We hypothesized that CDP-choline could reverse sepsis-induced microcirculatory alterations. Therefore this study determined the effects of CDP-choline on microvascular permeability and leukocyte-endothelial interactions during experimental endotoxemia.

## Methods

The general aspects of the materials and methods used have been described in detail in previous publications [4, 12].

### Anesthesia and animal preparation

The Governmental Animal Protection Committee approved the experimental protocol used in this investigation (project licence number 35–9185.81/G-123/10). Male Wistar rats (*n* = 39, randomized to study groups, 250–300 g body weight; Janvier; St Berthevin, France) were maintained in an animal facility with a 12-h light–dark cycle and housed in stainless steel cages in a temperature-and humidity-controlled room. Standard diet and water were available ad libitum.

Anesthesia was induced with 60 mg/kg pentobarbital i.p. (Narcoren®, Merial GmbH, Hallbergmoos, Germany). Anesthesia was maintained with repeated injections of 10 mg/kg pentobarbital i.v.. The left carotid artery was cannulated for continuous real time monitoring of mean arterial pressure (MAP [mmHg]) and heart rate (HR [min^−1^]) using a computerized non-commercial small animal monitoring system (Small Animal Monitoring Vs. 1.2.6.3, Exp. Chirurgie, Heidelberg 2012). The right jugular vein was cannulated for administrating test substances. Temperature was measured with a rectal thermistor probe and maintained at 37 °C. A segment of the ileum was gently exteriorized through a mid-line abdominal incision and draped over a clear glass pedestal for intravital microscopy examination. Exposed tissues were continuously superfused with buffered (pH 7.4) and thermostat-controlled Ringer solution (37 °C).

A stock solution of LPS (Lipopolysaccharide, *Escherichia coli* 026:B6, Sigma-Aldrich Chemie GmbH, Steinheim, Germany) was prepared by dissolving LPS in saline to a concentration of 5 mg/ml. The solution was stored in a glass container at 5 °C. The stock solution was diluted in saline to the appropriate concentration for experiments. Infusions of LPS (4 mg/kg/h) or equivalent volumes of saline solution were administered via the jugular vein. The LPS dosage of 4 mg/kg/h was identified in pilot experiments as the LPS dosage without pronounced hypotension and a significant inflammatory endothelial activation (macromolecular leakage, leukocyte-endothelial interactions) compared to nonendotoxemic animals.

**Fig. 1:**
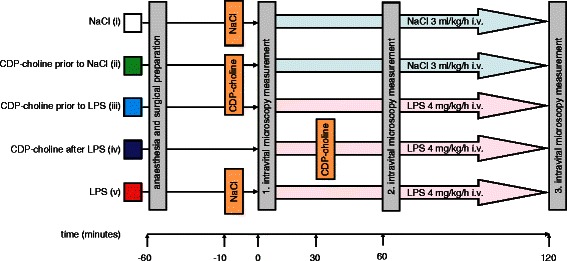
Experimental protocol. Intravital microscopic measurements (IVM) were performed at 0-, 60-, and 120- min in endotoxemic and nonendotoxemic animals following a stabilization period after surgical preparation. LPS (4 mg/kg/h) or an equivalent volume of saline was continuously infused starting directly after baseline IVM at 0 min. CDP-choline (100 mg/kg) was applied as an i.v. bolus in treatment groups. All administered fluids were calculated to guarantee that all animals received equal amounts of intravenous fluids. The color and number code of the experimental groups introduced in this figure is used in all other figures

Cytidine-5-diphosphocholine (CDP-choline, Sigma-Aldrich Chemie GmbH, Steinheim, Germany) was diluted in saline to the appropriate concentration for each rat and was injected i.v. according to the experimental protocol (Fig. 1).

For measurement of erythrocyte velocity fluorescent-labeled erythrocytes from donor rats were injected 10 min before baseline measurements (0.5 mL/kg body weight; hematocrit 50 %; labeled with a red fluorescent cell linker kit (PKH26-GL; Sigma Chemical, Deisenhofen, Deisenhofen, Germany).

To quantify albumin leakage across mesenteric venules, 50 mg/kg of fluorescein isothiocyanate-labeled bovine albumin (FITC-albumin; Sigma Chemicals, Deisenhofen, Germany) was injected 10 min before baseline measurements.

### Intravital microscopy

Intravital fluorescence microscopy (IVM) was performed at 0, 60, and 120 min (Fig. 1). Selected post-capillary venules were observed with a specially designed microscope (Orthoplan, Leica, Wetzlar, Germany) equipped with a 40-fold water immersion objective (Achroplan 40/0.75 W; Zeiss, Jena, Germany).

Images were recorded and digitized with a digital camera (TypPS/DX4-285FW, Kappa opto-electronics GmbH, Gleichen, Germany) equipped with a capturing software (Streampix 5.3.0, Norpix Inc., Montreal, Canada).

ImageJ (NIH, Bethesda, MD) and Histo (Histo, Version 3.0.2.4, Exp. Chirurgie, Uniklinik Heidelberg 2011) were used for offline image analysis.

The analysis software was calibrated to the aforementioned microscope/camera setting yielding a pixel spacing of 3.25 pixel/μm in effective magnification. A fluorescence gray scale from black to white (gray levels ranging from 0 [black] to 255 [white]) with fixed brightness and contrast levels was used for recording and analysis.

### Quantification of macromolecular leakage

The recorded fluorescent images were digitized and the gray levels reflecting fluorescent intensity were measured within the venule under study (iv) as well as in an equal and contiguous area of the perivenular interstitium (ii). Macromolecular leakage was determined as ii/iv ratio (arbitrary units).

### Leukocyte-endothelial interactions

The behavior of leukocytes was visualized using transillumination microscopy. Adherent leukocytes were defined as cells that did not move or detach from the endothelial wall for a period of 30 sec and were counted offline during playback of the recorded videos. Leukocyte adherence was expressed as the number of cells per square millimetre of vessel surface as calculated from the diameter and length of the vessel segment studied.

### Measurement of venular wall shear rate

Mean red blood cell velocities (V_RBC_) in single unbranched post-capillary venules were calculated by averaging the velocities of 20–30 individual erythrocytes. The distance through which a labeled erythrocyte travelled within two subsequent video frames was divided by the video frame time interval. Venular wall shear rate was calculated on the basis of the Newtonian definition (γ =8 (V_RBC_/D_v_)), using the measured vessel diameters of the observed postcapillary venules.

### Experimental protocol

**Fig. 2:**
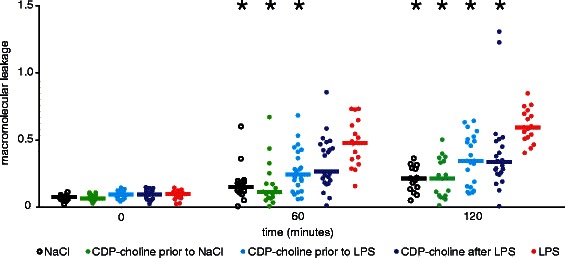
Effect of CDP-choline administration on macromolecular leakage during endotoxemia. Macromolecular leakage is expressed as ratio of perivenular to venular fluorescence intensity in arbitrary units. CDP-choline reduced microvascular permeability in postcapillary venules during endotoxemia. These results indicate that CDP-choline has a protective effect on microvascular permeability. Scatterplots with medians are displayed. *Significant difference vs. LPS (v). Medians with interquartile range (Q1–Q3) and p values are presented in Table 1

The experimental protocol is illustrated in Fig. 1. The color code of the experimental groups introduced in Fig. 1 is used in the results section for Fig. 2 and Fig. 4 as well.

Anaesthesia and surgical preparation was identical for all animals. A 45-min stabilization period followed exteriorization of the mesentery. Animals were randomized to the respective treatment groups 10 min before baseline intravital microscopic measurements (IVM) at 0 min. IVM were performed at 0, 60, and 120 min in endotoxemic and nonendotoxemic animals. Macrohemodynamic parameters (single values; MAP and HR) were documented at these time points. LPS (4 mg/kg/h) or an equivalent volume of saline was continuously infused starting directly after baseline IVM at 0 min. CDP-choline (100 mg/kg) was applied as an i.v. bolus in treatment groups. All administered fluids were calculated to guarantee that all animals received equal amounts of intravenous fluids.

After randomization animals [*n* = 39] received saline alone in the NaCl (i) [*n* = 8] group, CDP-choline 10 min prior to saline infusion (ii) [*n* = 6], CDP-choline 10 min prior to LPS administration (iii) [*n* = 8], CDP-choline 30 min after LPS administration (iv) [*n* = 9], and LPS alone (v) [*n* = 8] in the endotoxemia group.

At baseline IVM inclusion criteria for postcapillary venules were defined as a diameter of 25–35 μm, no more than 5 adherent leukocytes per 100 μm vessel length, no blood flow stasis, no lymph vessels or mesenterial fat tissue in the immediate vicinity of the observed vessel segment and at least one free vessel wall without concomitant or branching vessels.

In total 16 venules in the NaCl (i), 17 venules in the CDP-choline (ii), 22 venules in the CDP-choline (iii), 23 venules in the CDP-choline (iv), and 17 venules in the LPS (vi) groups fulfilled the criteria stated above and were recorded and analyzed.

### Statistical analysis

The main outcome parameter for this study was the perivenular macromolecular leakage around postcapillary venules. To evaluate the effect of CDP-choline on microvascular alterations during endotoxemia, all IVM-generated microvascular data are compared to the LPS group unless otherwise stated. The D'Agostino & Pearson omnibus normality test was applied to check for normal distribution. Due to nonparametric data distribution, nonparametric methods for evaluation were used (Wilcoxon test for differences within groups and Dunn's multiple comparisons test for differences between groups with multiplicity adjusted p values). Data were considered statistically significant at *p* < 0.05.

The results are presented in Tables 1, 2, 3 as median with interquartile range (Q1–Q3). Significant adjusted p-values of the comparisons with the LPS group and significant p-values of the comparisons with the baseline IVM measurement at 0 min are included in the tables.

**Table 1:** Summarized results of macromolecular leakage

		Time after induction of endotoxemia										
		0 min			60 min				120 min			
Macromolecular leakage [arbitrary units]		Median	IQR (Q1-Q3)	*	Median	IQR (Q1-Q3)	^*1^	#^2^	Median	IQR (Q1-Q3)	*	#
NaCI	(i)	0.075	0.05-0.09	ns^3^	0.15	0.11-0.2	<0.0001	0.0002	0.22	0.14-0.29	<0.0001	<0.0001
CDP-choline prior to NaCI	(ii)	0.067	0.05-0.09	ns	0.11	0.07-0.21	<0.0001	0.0054	0.21	0.08-0.36	<0.0001	0.0008
CDP-choline prior to LPS	(iii)	0.095	0.07-0.12	ns	0.24	0.12-0.42	0.0141	<0.0001	0.35	0.15-0.51	0.0019	<0.0001
CDP-choline after LPS	(iv)	0.095	0.06-0.12	ns	0.27	0.18-0.47	ns	<0.0001	0.34	0.25-0.49	0.0022	<0.0001
LPS	(v)	0.099	0.07-0.11		0.48	0.34-0.63		<0.0001	0.6	0.51-0.71		<0.0001

^1^ * significant adjusted p value vs. LPS (v)

^2^ # significant p value vs. 0 min

^3^ ns: not significant

**Table 2:** Summarized numbers of adherent leukocytes

		Time after induction of endotoxemia										
		0 min			60 min				120 min			
Adherent leukocytes [cells/ mm^2^]		Median	IQR (Q1-Q3)	*	Median	IQR (Q1-Q3)	*1	#^2^	Median	IQR (Q1-Q3)	*	#
NaCI	(i)	140	59-218	ns^3^	172	85-201	0.0139	ns	136	74-216	<0.0001	ns
CDP-choline prior to NaCI	(ii)	206	75-268	ns	204	108-251	ns	ns	220	147-337	0.015	ns
CDP-choline prior to LPS	(iii)	204	165-250	ns	243	169-375	ns	0.0175	322	234-423	ns	<0.0001
CDP-choline after LPS	(iv)	239	128-271	ns	305	232-351	ns	<0.0001	342	226-418	ns	0.0003
LPS	(v)	193	118-280		267	193-358		0.0038	403	224-562		<0.0001

^1^ * significant adjusted p value vs. LPS (v)

^2^ # significant p value vs. 0 min

^3^ ns: not significant

**Table 3:** Summarized macro- and microhemodynamic parameters

		Time after induction of endotoxemia										
		0 min			60 min				120 min			
Mean arterial pressure [mmHg]		Median	IQR (Q1-Q3)	*	Median	IQR (Q1-Q3)	*^1^	#^2^	Median	IQR (Q1-Q3)	*	#
NaCI	(i)	117	100-126	ns^3^	131	125-137	0.0037	ns	133	118-145	0.0386	ns
CDP-choline prior to NaCI	(ii)	108	101-118	ns	117	93-132	ns	ns	133	104-139	ns	ns
CDP-choline prior to LPS	(iii)	105	97-123	ns	105	101-114	ns	ns	113	60-117	ns	ns
CDP-choline after LPS	(iv)	106	94-120	ns	105	89-120	ns	ns	114	100-122	ns	ns
LPS	(v)	103	89-109		99	89-104		ns	106	97-112		ns
Heart rate [min^−1^]		Median	IQR (Q1-Q3)	*	Median	IQR (Q1-Q3)	*	#	Median	IQR (Q1-Q3)	*	#
NaCI	(i)	322	317-344	ns	371	332-403	0.0037	0.0002	395	326-441	0.0327	<0.0001
CDP-choline prior to NaCI	(ii)	358	338-411	ns	408	400-473	ns	0.0313	433	428-470	ns	0.0313
CDP-choline prior to LPS	(iii)	375	351-456	ns	475	447-566	ns	0.0156	449	441-493	ns	0.0313
CDP-choline after LPS	(iv)	353	287-380	ns	381	326-432	ns	0.0117	445	358-476	ns	0.0039
LPS	(v)	340	320-353		410	391-436		0.0078	491	435-516		0.0078
Venular wall shear rate [s^−1^]		Median	IQR (Q1-Q3)	*	Median	IQR (Q1-Q3)	*	#	Median	IQR (Q1-Q3)	*	#
NaCI	(i)	400	352-575	ns	505	413-642	ns	0.0006	444	368-573	ns	ns
CDP-choline prior to NaCI	(ii)	550	427-653	ns	420	356-614	ns	ns	302	230-631	ns	0.0395
CDP-choline prior to LPS	(iii)	436	367-567	ns	362	274-553	ns	0.0229	311	120-497	ns	0.0009
CDP-choline after LPS	(iv)	503	407-582	ns	397	321-505	ns	0.0301	443	281-590	ns	0.0254
LPS	(v)	499	405-622		378	251-523		ns	348	285-493		0.0026

^1^ * significant adjusted p value vs. LPS (v)

^2^ # significant p value vs. 0 min

^3^ ns: not significant

Statistical analysis was performed using GraphPad Prism version 6.0 for Mac OS X, GraphPad Software, San Diego California USA, www.graphpad.com.

## Results

### Macromolecular leakage

**Fig. 3:**
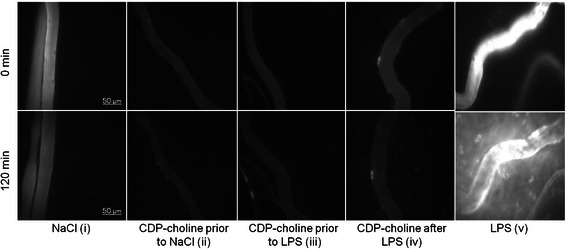
IVM images demonstrating the CDP-choline effect on microvascular permeability during endotoxemia. Fluorescent IVM images show postcapillary venules recorded at 0 min (upper panels) and 120 min after the treatment (lower panels). LPS administration alone (v) induces distinctive macromolecular leakage compared to the baseline measurement at 0 min and to the NaCl group (i). Note the effect of CDP-choline on reducing macromolecular leakage in endotoxemic animals (iii, iv) compared to the LPS group (v)

The baseline IVM measurement at 0 min revealed no significant differences in macromolecular leakage between all experimental groups. Macromolecular leakage increased significantly in all groups over 120 min compared to the respective baseline measurements with the highest rise of plasma extravasation in the LPS (v) group. Infusion of LPS (v) caused a significant increase in macromolecular leakage compared to the NaCl (i) group at 60 and 120 min. After 120 min all groups treated with CDP-choline showed a significantly reduced macromolecular leakage compared to the LPS (v) group. Table 1 summarizes the results of the IVM measurements of macromolecular leakage. Figure 2 illustrates the observed effect of CDP-choline on macromolecular leakage and displays the significant differences to the LPS (v) group. Representative IVM images are shown in Fig. 3.

### Leukocyte adherence

**Fig. 4:**
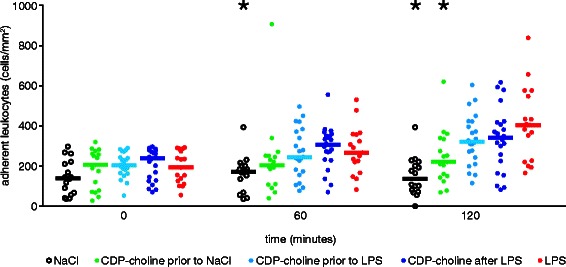
Effect of CDP-choline administration on leukocyte adherence during endotoxemia. CDP-choline had no effect on the number of adhering leukocytes during endotoxemia. Scatterplots with medians are displayed. * Significant difference vs. LPS (v). Medians with interquartile range (Q1–Q3) and p values are presented in Table 2

No significant differences were observed between all groups at the beginning of the experiment. The number of adhering leukocytes increased significantly over 120 min in all LPS treated groups (iii, iv, v) when compared to the respective baseline measurement. Leukocyte adhesion was significantly increased in the LPS group at 60 and 120 min compared to the NaCl (i) group and after 120 min compared to the nonendotoxemic CDP-choline (ii) group. No significant difference between the LPS (v) group and the endotoxemic CDP-choline (iii, iv) groups was observed during the experiment. CDP-choline administration without endotoxemia (ii) did not result in a significant increase in adhering leukocytes. In endotoxemic animals treated with CDP-choline (iii, iv) leukocyte adherence increased significantly compared to the baseline measurement during the experiment. Table 2 shows the summarized numbers of adherent leukocytes. Figure 4 illustrates the observed effect of CDP-choline leukocyte adhesion and displays the significant differences to the LPS (v) group.

### Macro- and microhemodynamic changes

There were no significant differences between all groups in the macro- and microhemodynamic parameters at the beginning of the experiment. The mean arterial pressure differed significantly between the NaCl (i) - and LPS (v) group at 60 min and at 120 min. No significant change in mean arterial pressure was observed within the groups. All groups showed a significant increase in heart rate over 120 min compared to the baseline measurement. Venular wall shear rate decreased significantly within the LPS group over 120 min. The venular wall shear rate decreased significantly after 120 min within all CDP-choline groups. The observed macro- and microhemodynamic changes are summarized in Table 3 including the significant changes compared to the IVM measurement at 0 min and to the LPS (v) group.

## Discussion

This study examined the effect of Cytidine-5-diphosphocholine (CDP-choline) on microcirculatory alterations during endotoxemia. LPS administration significantly increased microvascular permeability and leukocyte adherence and decreased venular wall shear rate in postcapillary venules. CDP-choline significantly reduced macromolecular leakage when applied before and after the start of the LPS infusion. In endotoxemic animals CDP-choline did not affect the number of adherent leukocytes and the reduced venular wall shear rate observed after LPS treatment.

The key result of this study is that CDP-choline has a protective effect on microvascular permeability during experimental endotoxemia.

Non-neuronal cholinergic mechanisms are fundamental processes for the fine-tuning of endothelial hemoeostasis [13]. Both direct and indirect cholinergic stimulation exhibit anti-inflammatory effects on endothelial activation and leucocyte-endothelial interactions [3, 4, 14]. Proinflammatory endothelial cell activation can be suppressed by the activation of specific nicotinic acetylcholine receptors like the α7nAChR [15] associated with the cholinergic anti-inflammatory pathway [5, 6, 16]. CDP-choline had a protective effect on microvascular barrier function when applied before and after the onset of endotoxemia in this study. Intravenous administration of CDP-choline increases plasma and brain choline and acetylcholine levels in rats [17, 18]. Choline as a selective natural α7nAChR agonist improves survival in experimental sepsis [19]. Gurun et al. demonstrated that localized administration of CDP-choline reduced tissue edema and TNF-α production in a α7nAChR-dependent mechanism [11]. Moreover activation of central cholinergic transmission by CDP-choline has protective α7nAChR- independent effects on myocardial ischemia-reperfusion damage [18].

The observed protective effect of CDP-choline on microvascular permeability is therefore probably attributable to a cholinergic activation of endothelial cells that could be directly mediated by α7nAChR on a cellular level or by central anti-inflammatory cholinergic signals. This protective effect is in line with scientific evidence regarding the anti-inflammatory potential of CDP-choline in other experimental settings [20–23].

There is ample evidence for interdependent leukocyte-dependent and leukocyte-independent mechanisms affecting endothelial permeability [12, 24]. Leukocyte recruitment can be blocked by α7nAChR-dependent cell activation [3].

In contrast to the effect of the centrally acting acetylcholinesterase inhibitor physostigmine, which reduced leukocyte adherence [4], CDP choline did not affect leukocyte adhesion during endotoxemia. This could indicate that leukocyte activation is more affected by centrally mediated cholinesterase inhibition or by more effectively elevating ACh serum levels. CDP-choline and choline are α7nAChR agonists with a lower potency as ACh, possibly explaining the missing effect on the numbers of adherent leukocytes observed in this study. LPS causes shedding of the endothelial glycocalix in rats [25], and inhibition of glycocalyx shedding reduces leukocyte-endothelial adhesion [26]. Increased microvascular permeability due to a breakdown of the endothelial glycocalix results in an increased transcapillary escape rate of albumin in experimental sepsis [27]. The decreased macromolecular leakage of FITC-albumin in the endotoxemic CDP-choline treated groups could be interpreted as a stabilization of the endothelial surface layer. A modulation of glycocalix function influencing the interaction between endothelial cells and leukocytes could be an explanation for the observed protective effect of CDP-choline on microvascular permeability paralleled by increased numbers of adherent leukocytes in the CDP-choline treated endotoxemic groups. Further studies focusing on qualitative effects of CDP-choline on leukocyte-endothelial interactions and glycocalix function are required to evaluate the in vivo findings of this study. The increased leukocyte adherence in LPS-treated animals is consistent with the decreased venular wall shear rate induced by endotoxemia [28]. Venular wall shear rate decreased significantly in all CDP-choline treated groups. Interestingly the largest decrease in venular wall shear rate was observed within the nonendotoxemic CDP-choline treated group indicating a direct effect of CDP-choline on the microcirculation without causing increased microvascular permeability and leukocyte adherence. In LPS treated animals CDP-choline treatment had no distinguishable effect on venular wall shear rate. Microvascular flow is impaired during early sepsis and uncoupled from macrovascular function [28]. This uncoupling is clearly demonstrated by the hemodynamic profile of the LPS (v) group. Macrohemodynamic parameters were not distinguishably affected by CDP-choline treatment. It has been demonstrated that CDP-choline restores renal and mesenteric arterial blood flow and hypotension in shock states and prevents death in myocardial ischaemia–reperfusion injury by activating central cholinergic neurotransmission [17, 18, 29]. This indicates that CDP-choline has positive effects on a compromised circulatory system. We did not induce an endotoxemic shock during the 120 min observation time of this study therefore no final conclusion can be drawn regarding the hemodynamic effects of CDP-choline. Given the heterogeneity of microcirculatory perfusion during sepsis, microvascular changes in the mesenteric postcapillary venules examined may not reflect the microcirculatory situation in other vascular beds. Therefore further studies to evaluate the hemodynamic and microcirculatory effects of CDP-choline during sepsis remain to be done.

### Limitations

We could not quantify plasma cytokines and glycocalyx marker in blood serum samples of the experimental animals due to the interference of the fluorescent dyes used for IVM with the standardized ELISA technology. Therefore, no direct correlation between our IVM results and the effect of CDP-choline on the systemic inflammatory response and the degree of glycocalyx shedding could be drawn.

## Conclusion

CDP-choline was effective in reducing LPS-induced microvascular permeability in endotoxemic rats. Leukocyte adherence and venular wall shear rate were not affected by CDP-choline during endotoxemia. Regarding the effect of CDP-choline on leukocyte-endothel interactions and microcirculatory alterations in this study, no final conclusion can be drawn necessitating further studies.

The protective effect on microvascular permeability of CDP-choline in this study may carefully be used as a basis for a clinical approach to stabilize capillary leakage in human sepsis. CDP-choline has been thoroughly studied in clinical trials for its brain protective properties on cerebrovascular and neurodegenerative diseases with an excellent drug safety profile [8–10, 30]. Considering its anti-inflammatory efficacy CDP-choline is a substance with great potential for clinical sepsis treatment.
